# Successful removal of a giant esophageal lipoma by thoracoscopic enucleation: a case report

**DOI:** 10.1186/s40792-020-0782-7

**Published:** 2020-01-13

**Authors:** Wataru Goto, Katsunobu Sakurai, Naoshi Kubo, Yutaka Tamamori, Kiyoshi Maeda, Masaichi Ohira

**Affiliations:** 1 Department of Gastroenterological Surgery, Osaka City General Hospital, 2-13-22 Miyakojimahondori, Miyakojima-ku, Osaka, 534-0021 Japan; 2 Department of Surgical Oncology, Osaka City University Graduate School of Medicine, 1-4-3 Asahi-machi, Abeno-ku, Osaka, Japan

**Keywords:** Video-assisted thoracoscopic surgery, Esophageal lipoma, Enucleation

## Abstract

**Background:**

We report a rare case of giant esophageal lipoma treated with thoracoscopic enucleation successfully.

**Case presentation:**

A 69-year-old woman was referred to our department with dysphagia. Computed tomography examination revealed a large hypoattenuating submucosal mass with dense fat in the middle and lower esophagus. Upper gastrointestinal endoscopy revealed a submucosal mass with normal mucosa on the middle and lower esophageal wall. On a diagnosis of esophageal lipoma, we performed a video-assisted thoracoscopic operation and the 14.0 × 6.5 × 3.0 cm in size submucosal tumor was completely enucleated. We could successfully avoid a subtotal esophagectomy with high invasiveness. The patient was discharged on the 36th day after operation, and no symptoms had been noted.

**Conclusions:**

Video-assisted thoracoscopic enucleation with minimal invasiveness may be an appropriate treatment option even for such a huge benign esophageal submucosal tumor.

## Background

Benign tumors of the esophagus are very rare. Esophageal lipomas, in particular, account for only 0.4% of all digestive tract benign tumors [1]. Because most esophageal lipomas are small and asymptomatic, many cases are found incidentally during imaging studies. Rarely, esophageal lipomas become large and tend to produce symptoms such as dysphagia, and surgical excision is required. Herein, we report a rare case of giant esophageal lipoma treated with thoracoscopic enucleation successfully.

## Case presentation

**Fig. 1:**
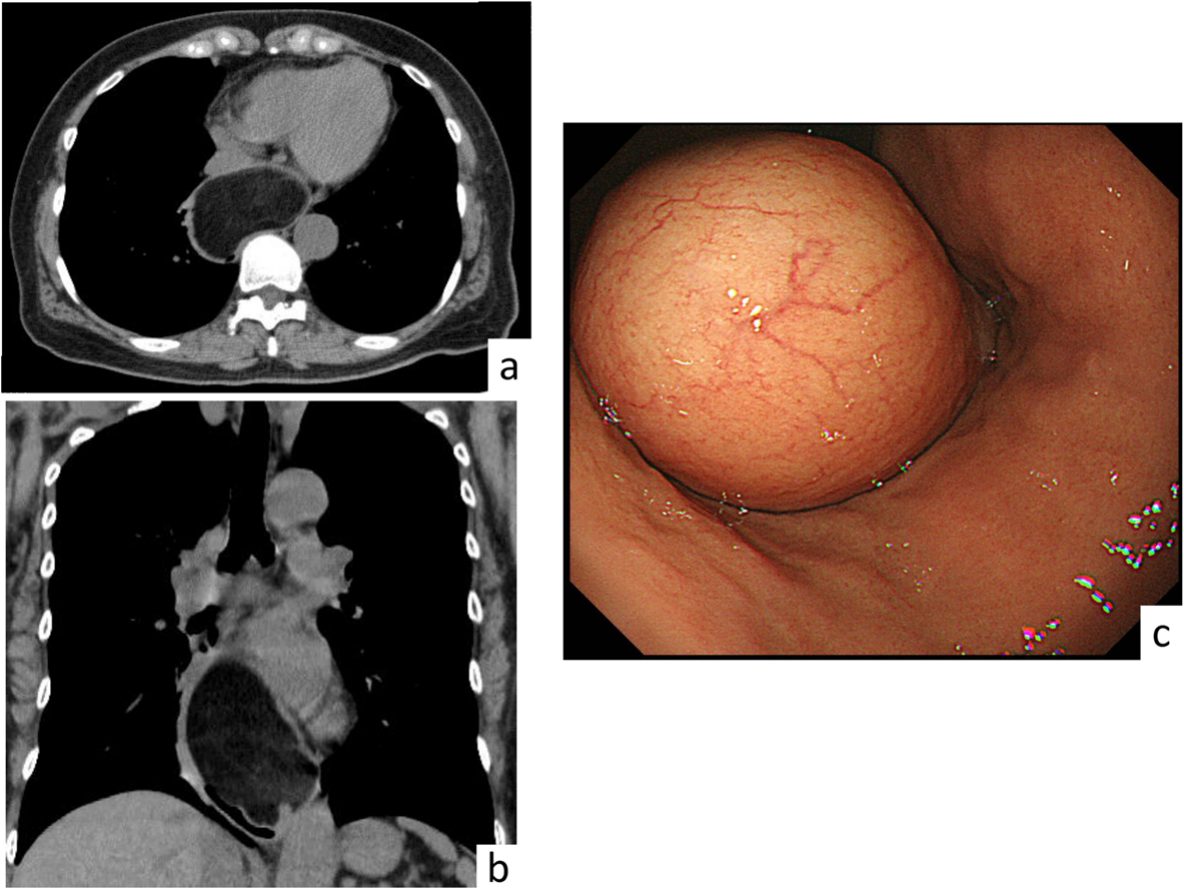
Preoperative imaging. (**a** horizontal, **b** coronal) CT scan of the chest revealed a 10 × 7 cm homogenous mass in the middle and lower esophagus. **c** Upper endoscopy revealed a submucosal tumor with normal mucosa arising from the left esophageal wall

A 69-year-old woman was admitted to our hospital with dysphagia during the previous 1 month. The clinical interview disclosed a history of uterine myoma and bronchial asthma. Physical examination on admission revealed normal findings. A chest computed tomography (CT) scan revealed a 10 × 7 cm submucosal mass in size on the middle and lower esophagus. Upper border of the mass was not close to the tracheal bifurcation. The tumor was hypoattenuating and characteristic of adipose tissue (Fig. 1a,b). Upper gastrointestinal endoscopy revealed a submucosal mass with normal mucosa, arising from left side of the middle and lower esophageal wall. The tumor was soft, and endoscopy could easily pass through the esophageal lumen (Fig. 1c). Fluorodeoxyglucose (FDG) positron emission tomography (PET) revealed an adipose tissue-like mass of the middle and lower esophagus without FDG accumulation. Tumor markers were within normal limits, squamous cell carcinoma antigen was 0.7 ng/ml, and cytokeratin 19 fragment was 1.4 ng/ml. Thus, we diagnosed with left esophageal lipoma, and video-assisted thoracoscopic enucleation of the esophageal lipoma was performed. Though the tumor was located on the left side of the esophageal wall, it was very large, and the esophagus was greatly shifted to the right side of the thoracic cavity. Thus, we selected a right-side transthoracic approach in accordance with our usual manner of esophagectomy.

**Fig. 2:**
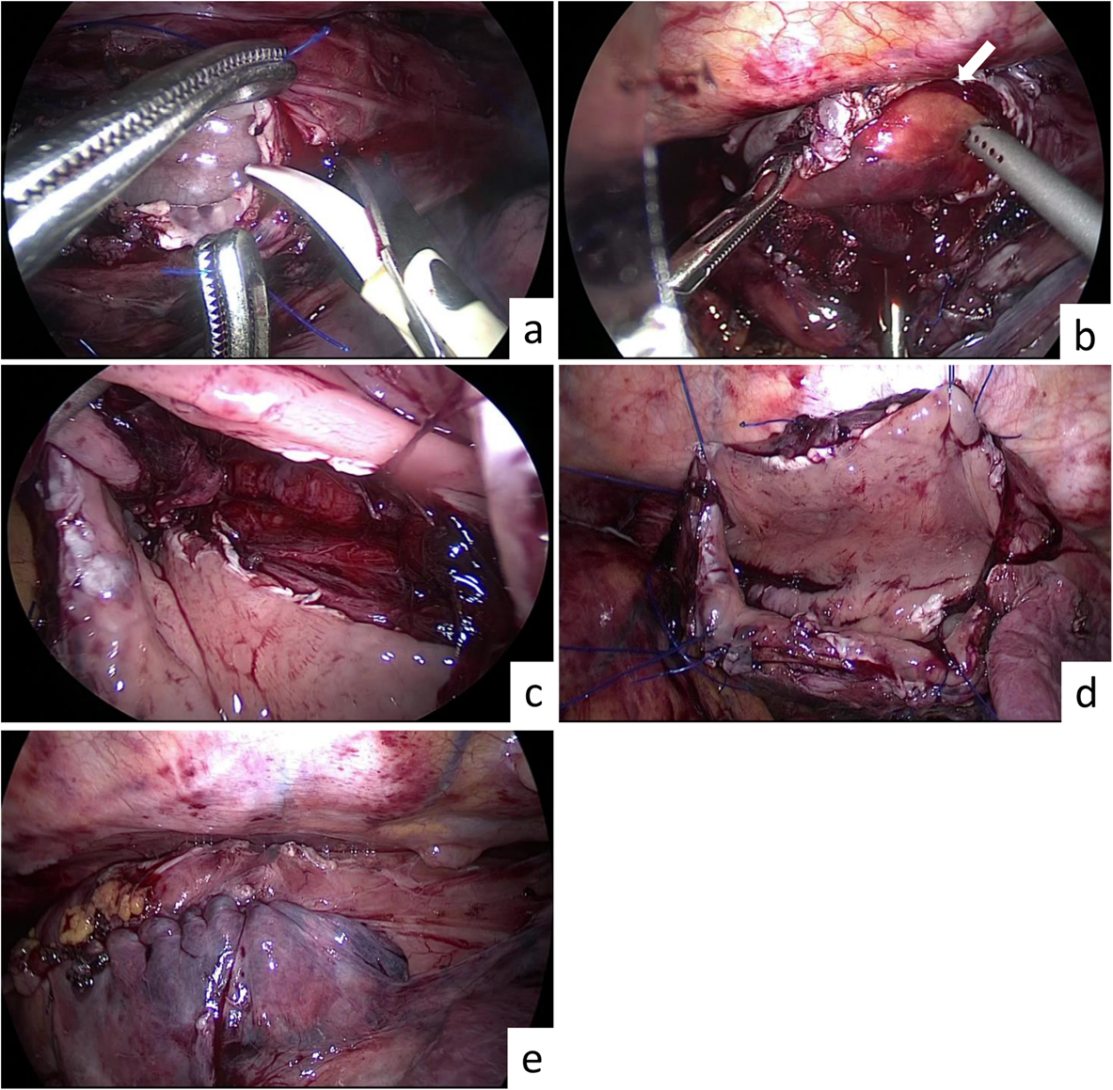
Thoracoscopic enucleation of a giant esophageal lipoma. **a** A longitudinal incision of the right esophageal wall was performed. **b** A longitudinal incision of the mucosa of the left esophageal wall was performed, and a lipoma (arrow) started to rise. **c** After enucleation. Muscle layer was not injured. **d** Four retraction sutures were placed on esophageal wall and held by an assistant. **e** After suturing the mucosa and esophageal wall

**Fig. 3:**
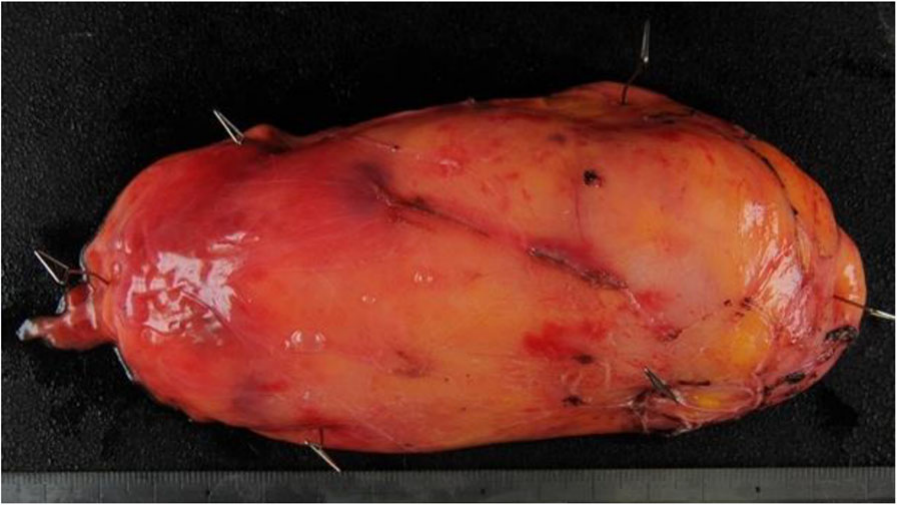
Histopathological examination. The yellow and soft giant lipoma, measuring 14.0 × 6.5 × 3.0 cm in size with diffuse mature adipose tissue

The patient was placed in a left half prone position in accordance with thoracoscopic surgery for esophageal cancer in our department. Two 12 mm ports were inserted at 6th intercostal space (ICS) at the anterior axillary line for assistant and 7th ICS at the posterior axillary line for scope, and two 5 mm ports were inserted at 5th and 9th ICS at the midaxillary line in the right lateral chest wall respectively for operator. First of all, the mediastinal pleura was opened, and a large bulge was visualized in the middle and lower esophagus. The esophagus was mobilized circumferentially, and two 3-0 monocryls were placed around the middle and lower esophagus to retract the esophagus for the remainder of the procedure. A longitudinal incision of the right esophageal wall was performed to expose the lumen of esophagus (Fig. 2a). Then, by a longitudinal incision of the mucosa of the left esophageal wall, the yellow, soft, and smooth submucosal tumor was confirmed (Fig. 2b). The tumor was separated gradually from the surrounding mucosa and muscle with a blunt dissection and usage of vessel sealing system without any perforation of esophageal wall, and an enucleation was completed (Fig. 2c). After confirmation that muscle layer of the left esophageal wall was not injured, four retraction sutures were placed on the esophageal wall and retracted by an assistant (Fig. 2d). The split mucosa layer of the left esophageal wall was repaired with barbed suture. Then, the right esophageal wall opening was closed with layer to layer using barbed sutures as well. No stenosis and perforation of the esophageal wall was confirmed by upper gastrointestinal endoscopy, and the mediastinal pleura was repaired (Fig. 2e). Subsequently, a 24-Fr chest tube was left, and the giant lipoma was extracted through the expanded wound of 12 mm port at the anterior axillary line. The total operation time was 335 min, and the blood loss was 150 ml. The macroscopic evaluation showed a well-capsuled, yellow, and soft 14.0 × 6.5 × 3.0 cm mass in size, and histopathological examination revealed diffuse mature adipose tissue, compatible with the diagnosis of esophageal benign lipoma (Fig. 3).

**Fig. 4:**
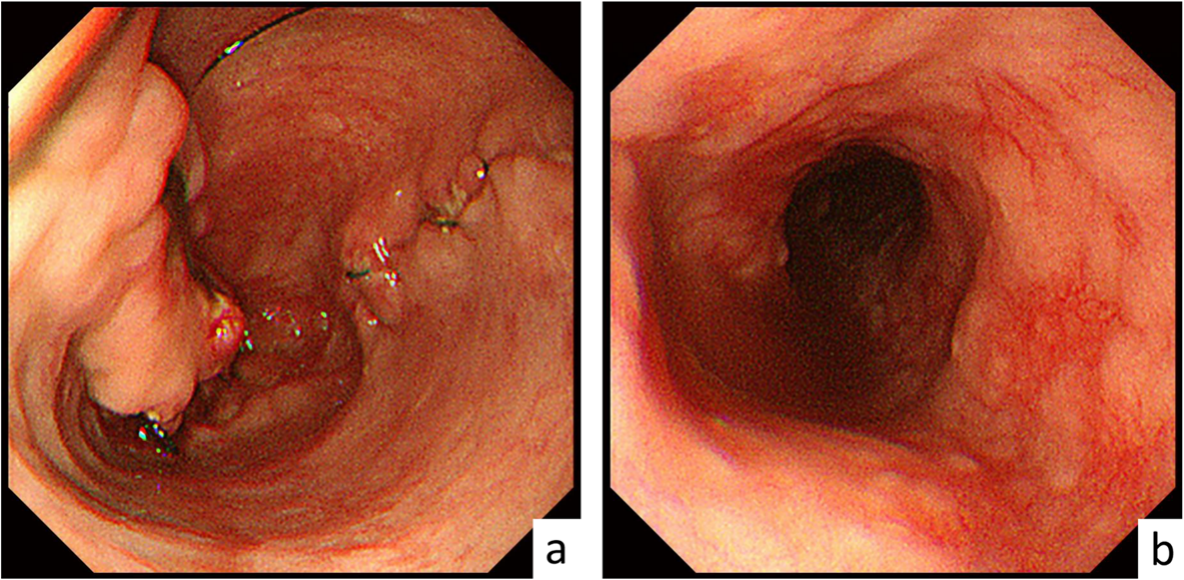
Upper endoscopy 8 days after the operation. **a** Anastomotic ulcer. **b** Reflux esophagitis

Upper gastrointestinal endoscopy 8 days after the operation showed anastomotic ulcer and reflux esophagitis (Fig. 4a). These were gradually improved by administration of omeprazole. In addition, the amidotrizoic acid swallow demonstrated dysperistalsis of the stomach on the 18th day; hence, fasting and infusion treatment were performed for a while. The patient gradually started eating more and was discharged on the 36th day after operation. Since then, no symptoms had been noted.

## Discussion

Esophageal submucosal tumors represent less than 1% of all esophageal neoplasms [2]. Of these lesions, leiomyomas account for 70–80%. Esophageal lipomas are very rare, accounting for 0.4% of benign tumors of the alimentary tract and usually occur from the cervical and upper esophagus, and are more common in men than in women [3]. On CT examination, lipomas show homogenous lesions containing dense fat. In addition, a smooth surface and “squeeze sign” manifested by deformation in contour and configuration as a result of peristalsis are useful to differentiate lipomas from other benign and malignant lesions [3]. Most esophageal lipomas are small and occur solitary; they do not cause symptoms and may be found incidentally during imaging studies. However, lipomas over 2 cm in diameter tend to cause symptoms such as dysphagia or odynophagia and require treatment. Various treatment options are available, depending on the tumor location and size, and include excision by endoscopy or esophagectomy [4]. However, esophagectomy for benign tumors is advocated as a high invasiveness, and it is important to select a procedure with low risk of morbidity as much as possible. Though enucleation is a useful treatment for esophageal lipomas, injury of esophageal wall during this procedure is a cause of conversion to esophagectomy, so separating the submucosal tissue should be done carefully [5]. In addition, minimally invasive surgery such as thoracoscopic or laparoscopic is related to reduction of postoperative pneumonia and shorter hospital stay compared with open approach [6]. Most large submucosal tumors of the esophagus are removed by thoracotomy [6]. However, because lipomas have well-defined, smooth surface and are quite soft, it was easy to separate the tissue around the tumor and to extract the tumor from small thoracoscopic wound. So, even a giant tumor like our case could be removed by thoracoscopy.

There are few case reports of esophageal lipoma treated with thoracoscopic enucleation. Though Chien-Ying Wang et al. [7] reported a case of esophageal lipoma removed by thoracoscopic enucleation, the maximum diameter of the tumor was 3.3 cm, which was quite small compared with our case. If a small submucosal tumor is located on the left wall of the esophagus, it is considered possible to perform minimally invasive surgery by enucleation with the left-side transthoracic approach. In our case, the tumor was located on the left side of the esophageal wall. However, since the lipoma was very large and the esophagus was greatly shifted to the right side of the thoracic cavity, the possibility of conversion to esophagectomy was considered. Therefore, we selected a right-side transthoracic approach according to our usual manner of esophagectomy, which can respond to the change of surgery procedure.

The knack of this operation is to develop the appropriate submucosal layer along the tumor surface to preserve the muscle layer, after incision of mucosa of left esophageal wall. Since the tumor was elastic soft, the enucleation was possible with a mucosal incision shorter than the actual tumor size. In addition, after a longitudinal incision of the right esophageal wall, by four retraction sutures placed on the esophageal wall from the outside of the thorax to secure the view of esophageal lumen, we could easily separate the submucosal layer or reapproximate split mucosa layer (Fig. 2d). If the injury or stenosis of the esophageal wall is concerned during thoracoscopic enucleation, esophagectomy and esophagogastrostomy are unavoidable. However, in our case, no stenosis and perforation of the esophageal wall was confirmed by intraoperative endoscopy after suturing. Based on this case, thoracoscopic enucleation with right-side transthoracic approach was considered one of the treatment options even for the giant lipoma located on the left esophageal wall.

However, video-assisted thoracoscopic enucleation and suture can be performed safely only by well-trained surgeons. Watanabe et al. reported the difficulty of thoracoscopic enucleation for an esophageal schwannoma larger than 5 cm and the conversion to subtotal esophagectomy [8]. Recently, the robotic approach for enucleation of esophageal lipoma was reported [9]. Because the robot-assisted surgery offers advantages, including wrist-like movement of the instruments, the three-dimensional camera, and hand tremor filtration, the robotic approach is likely to make surgical procedures, such as tissue dissection and suture, easier.

In addition, there are other suggested surgical plans for enucleation of esophageal lipoma. Tsalis et al. [4] reported that a Nissen fundoplication was performed to reinforce the esophageal wall and to prevent reflux esophagitis after laparoscopic enucleation of giant lipoma of the lower esophagus, and Jeon et al. [10] reported that intraoperative esophagoscopy assistance was useful to identify accurate localization of small esophageal submucosal tumors and to evaluate whether there was perforation or stenosis of the esophageal wall after enucleation. Further developments of safe and useful treatment are expected.

## Conclusions

We could remove an exceptionally large esophageal lipoma with thoracoscopic enucleation. Although esophageal lipomas are extremely rare, thoracoscopic enucleation is considered a safe and useful treatment.

## Data Availability

Data sharing is applicable to this article.

## Abbreviations

CT: Computed tomography
FDG: Fluorodeoxyglucose
ICS: Intercostal space
PET: Positron emission tomography
